# Insights into Network of Hot Spots of Aggregation in Nucleophosmin 1

**DOI:** 10.3390/ijms232314704

**Published:** 2022-11-25

**Authors:** Daniele Florio, Sara La Manna, Concetta Di Natale, Marilisa Leone, Flavia Anna Mercurio, Fabiana Napolitano, Anna Maria Malfitano, Daniela Marasco

**Affiliations:** 1 Department of Pharmacy, University of Naples “Federico II”, 80131 Naples, Italy; 2 Department of Chemical, Materials and Production Engineering, University of Naples “Federico II”, 80125 Naples, Italy; 3 Institute of Biostructures and Bioimaging (CNR), 80145 Naples, Italy; 4 Department of Translational Medical Science, University of Naples “Federico II”, 80131 Naples, Italy

**Keywords:** aggregation, ThT fluorescence, bioinformatic prediction

## Abstract

In a protein, point mutations associated with diseases can alter the native structure and provide loss or alteration of functional levels, and an internal structural network defines the connectivity among domains, as well as aggregate/soluble states’ equilibria. Nucleophosmin (NPM)1 is an abundant nucleolar protein, which becomes mutated in acute myeloid leukemia (AML) patients. NPM1-dependent leukemogenesis, which leads to its aggregation in the cytoplasm (NPMc+), is still obscure, but the investigations have outlined a direct link between AML mutations and amyloid aggregation. Protein aggregation can be due to the cooperation among several hot spots located within the aggregation-prone regions (APR), often predictable with bioinformatic tools. In the present study, we investigated potential APRs in the entire NPM1 not yet investigated. On the basis of bioinformatic predictions and experimental structures, we designed several protein fragments and analyzed them through typical aggrsegation experiments, such as Thioflavin T (ThT), fluorescence and scanning electron microscopy (SEM) experiments, carried out at different times; in addition, their biocompatibility in SHSY5 cells was also evaluated. The presented data clearly demonstrate the existence of hot spots of aggregation located in different regions, mostly in the N-terminal domain (NTD) of the entire NPM1 protein, and provide a more comprehensive view of the molecular details potentially at the basis of NPMc+-dependent AML.

## 1. Introduction

Amyloids can be divided into three main groups [1]: (1) pathological amyloids, which were the first to be discovered [2,3]; (2) artificial amyloids, often deriving from natural or de novo conceived sequences [4,5,6]; (3) naturally occurring functional amyloids, which perform a wide range of biological functions in diverse organisms (bacterial biofilms [7], scaffolding for melanin synthesis [8], storing peptide hormones [9]), including the formation of protein complexes in subcellular condensates [10,11,12]. In neurodegeneration [13], pathological aggregates can form amorphous assemblies [14] and/or highly ordered cross-β amyloid fibers [15]; the toxic species are often small, disordered oligomers, as precursors of fibrils [16]. To unveil the basis of toxicity of soluble aggregated, proto- and mature fibrils, it is of fundamental interest to deepen the mechanisms of fibrillogenesis. The successful prediction of the aggregation propensity of amino acidic sequences helps investigate the amyloid process [17]. Hence, the identification of short protein stretches, called the aggregation-prone regions (APR), is a powerful reductionist approach, opposite to the experimental complexity due to protein length, composition and concentration [18,19]. The self-assembly of APRs is modulated by both homo- and heterotypic interactions, as recently demonstrated among Aβ stretches [20] and for α-synuclein and tau K18 proteins; for them, electrostatic interactions between the negatively charged C-terminal segment of α-synuclein and the positively charged tau K18 fragment occurred [21]. Consistent with this “amyloid stretch hypothesis”, many computational algorithms are used to search for the fragments predicted as amyloidogenic, with different success rates [22,23,24]. Within cells, membraneless organelles composed of proteins and nucleic acids exert primary roles [25,26]. They are viscoelastic aggregates with diverse properties and destinations [27] and dynamic and reversible formations via liquid–liquid phase separation (LLPS) [28]. Globular and amyloid states are thermodynamically correlated in the conformational effects of point mutations in proteins [28]. For example, p53 aggregation can lead to loss-of-function (LoF), dominant-negative (DN) and gain-of-function (GoF) effects, with adverse cancer consequences [29]. Nucleophosmin (NPM)1 protein is a crucial regulator of p53, and the alteration of NPM1′s functions can concur with the dysregulation of p53 in tumors [30]. More generally, NPM1 is the major granular component of the nucleolus [31] and shuttles rapidly between the nucleus and cytoplasm in its chaperonin role [32].

NPM1 has a modular structure (Figure S1) with three main functional and structural domains [33,34,35,36]. The experimental crystal structure of the N-terminal domain (NTD) exhibited eight β-barrels forming a jelly roll barrel; monomers associate to form pentamers, which, in turn, can interact to form a decamer, indicating a certain structural plasticity at the pentamer–pentamer interface [37]. The NTD-dependent oligomeric states of NPM1 are characterized by different and numerous post-translational modifications, especially phosphorylation, which modulate protein localization and function [38]. The NPM1 central portion is predicted as unstructured (intrinsically disordered region, IDR) and is marked by the presence of highly acidic regions (A2 and A3) rich in aspartic and glutamic acids. Pentameric NPM1 undergoes LLPS via heterotypic interactions with nucleolar components, including ribosomal RNA (rRNA) and proteins displaying multivalent arginine-rich linear motifs (R-motifs); however, it also forms homotypic interactions among its polyampholytic IDR [38,39,40,41,42]. The C-terminus of NPM1 (CTD) in its globular form experimentally exhibited a three-helix-bundle tertiary structure [43] and was characterized by the presence of a basic, positively charged cluster of amino acids, immediately followed by a stretch of aromatic residues, providing an adequate platform allowing the binding to nucleic acids and ATP [34,44]. These aromatic residues constitute an atypical nucleolar localization signal (NoLS), and their mutations cause the unfolding of the CTD and the aberrant protein cytoplasmic localization typical of acute myeloid leukemia (AML) cells (NPMc+). The intra [45] and inter [40] domain interactions deeply modulate the thermodynamic stability of the entire protein, its nucleolar retention and RNA-binding properties. Our recent studies highlighted an unexpected propensity for amyloid aggregation of several regions of NPMc+ CTD, often providing cytotoxic species [46,47,48,49,50,51,52,53,54,55,56,57]. On the basis of the mutual influence of protein domains already observed for NPM1 [40,45], in this study, we investigate the existence of hot spots of aggregation in the entire protein, including NTD and IDR. By following a complementary theoretical and experimental approach, several protein regions prone to aggregation were predicted, and this ability was investigated by means of ThT fluorescence, circular dichroism and NMR spectroscopies, and the related aggregates were analyzed by SEM. A few protein regions were also assayed in SHSY5 cells.

## 2. Results and Discussion

- Structure prediction and aggregation propensity through bioinformatic tools.

The amyloid propensity of a great number of proteins depends on the presence and collocation of APRs. Often, flexible segments belonging to IDRs, even if located away from APRs, can act as conformational wings in the formation of amyloid assemblies. Aggregation predictor algorithms aim to “read” the aggregation propensities from the primary sequence even if, during folding, the APRs can be protected by chaperones and self-chaperoning interactions [5].

Our recent results demonstrate, unexpectedly and unequivocally, that the CTD of NPM1 in AML mutated forms are prone to aggregate. In the present study, we aimed to identify new hot spots of aggregation on the whole primary sequence (1–294 residues) of NPM1.

**Figure 1:**
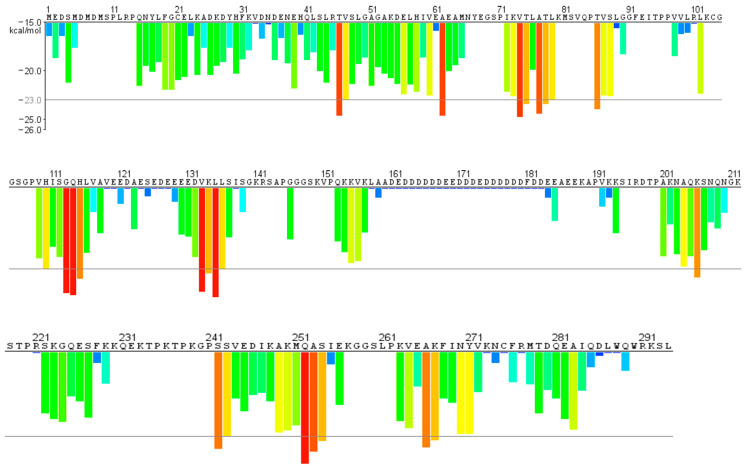
Zippedb predictions of the entire NPM1_1–294_ primary sequence.

For this purpose, we analyzed the protein sequence through https://services.mbi.ucla.edu/zipperdb/intro. Figure 1 presents a plot with the primary sequence of NPM1 on the X-axis with a histogram bar proportional to the Rosetta energy of each residue. The orange-red segments with energy values below the indicated threshold of −23 kcal/mol (gray line) are predicted to form fibrils. Hence, we designed, ad hoc, six peptides covering the protein fragments reported in Table 1. These regions were conceived to both contain red histograms–residues and defined secondary structures. The conformational knowledge derived from known experimental structures of separated domains, NTD (1–117 residues) [37] and CTD (243–294 amino acids) [43], which were further confirmed by using the prediction server PSIPRED (http://bioinf.cs.ucl.ac.uk/psipred/) for the entire 1–294 protein (Figure S2). Thus, during the design process, two main factors were considered: (i) the restriction to the minimum fragment containing hot spots of aggregation and (ii) the presence of defined secondary structures, providing fragments with very different lengths. In them, to avoid the “extremity effects” of protein dissection, the predicted amyloid stretch was located at the center of the dissected regions. Noticeably, from this analysis, the protein fragment 242–259, corresponding to the first helix of the three-helix bundle (H1), emerged. This fragment was already investigated by us for its helical features [58] but not for the amyloidogenic propensity, and thus, we included it in the present study. Conversely, in the second helix of the bundle (H2 region), the fragment centered on the 269–277 stretch was outlined from amyloidogenic prediction (Figure 1). In this case, we did not include it in the present study, since this region was subject to many previous investigations [59].

In the NTD, an extended β-sheet structure is present (Figure S1) [37]. Herein, we selected several fragments centered on the hot spots of aggregation: 41–65 on Thr^46^, Ala^62^, 69–83 on Val^74^, Thr^75^ Ala^77^, Thr^78^ and 107–120 on Gly^113^, Gln^114^, His^115^. On the other hand, fragment 84–93, with Thr^86^ as the amyloidogenic residue, was designed as a random fragment in accordance with both experimental evidence and bioinformatics predictions. In the end, fragment 127–139 with Val^132^, Lys^133^ Leu^134^ was predicted to be endowed with helical conformation, while 242–259, bearing Gln^252^, Ala^253^, Ser^254^ as spots, covered the H1 helix [43] (Figure 1 and Figure S2). The peptide sequences and conformations (predicted and experimental) are reported in Table 1.

All sequences were synthesized, in the acetylated and amidated form, by SPPS with discrete yields using Fmoc methodologies and purified as already reported [47].

- β-sheet regions: ThT assay, conformational and SEM analyses of NPM1_41–65_, NPM1_69–83_ and NPM1_107–120._

**Figure 2:**
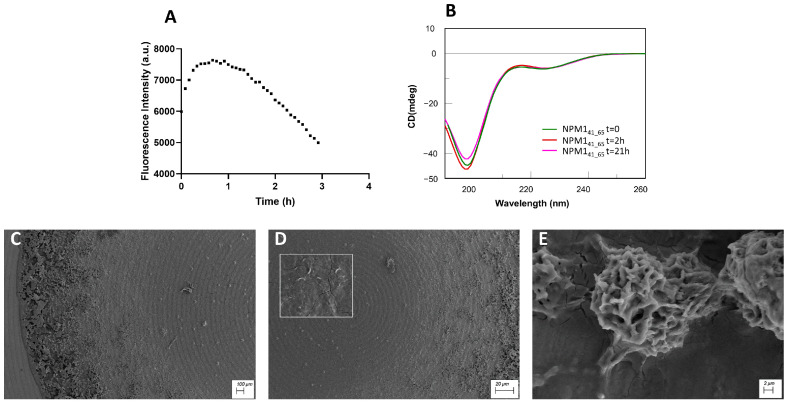
NPM1_41–65_ characterization: (**A**) Time course of ThT fluorescence emission intensity; (**B**) CD spectra over time; (**C**–**E**) SEM micrographs registered at 24 h at 100, 20 and 2 µm.

**Figure 3:**
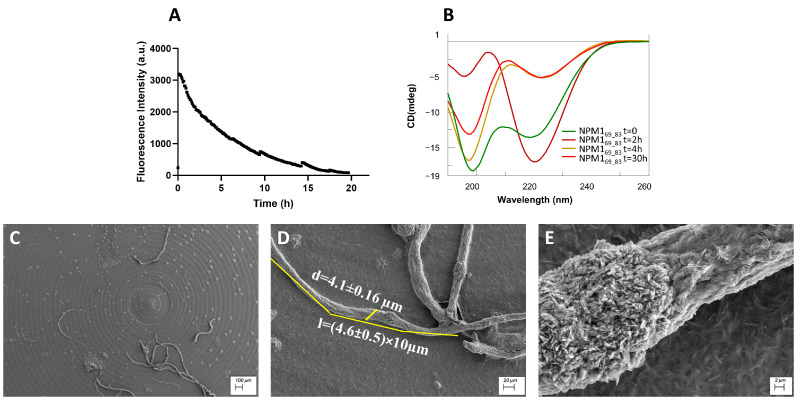
NPM1_69–83_ characterization: (**A**) Time course of ThT fluorescence emission intensity; (**B**) CD spectra over time; (**C**–**E**) SEM micrographs registered at 24 h at 100, 20 and 2 µm.

**Figure 4:**
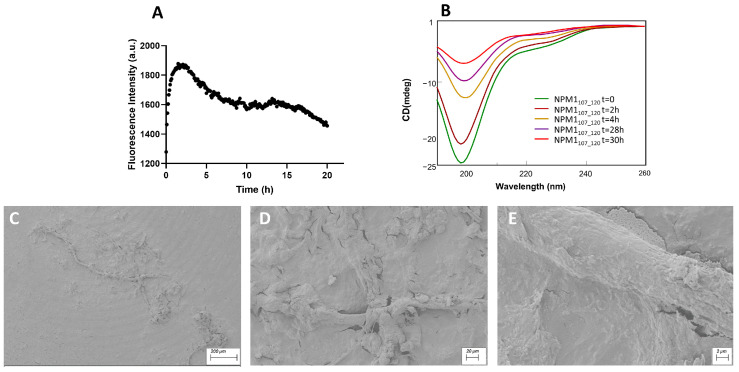
NPM1_107–120_ characterization: (**A**) Time course of ThT fluorescence emission intensity; (**B**) CD spectra over time; (**C**–**E**) SEM micrographs registered at 24 h at 200, 20 and 2 µm.

The peptides corresponding to regions 41–65 (Figure 2), 69–83 (Figure 3) and 107–120 (Figure 4) were firstly analyzed for their ability to bind ThT dye. The NPM1_69–83_ peptide appeared already aggregated at t = 0 (Figure 3A) with a very slow signal decrease; noticeably, this sequence is the only one to present a positive net charge at neutral pH (Table 1). NPM1_41–65_ also appeared partially aggregated at t = 0, suggestive of the presence of low order aggregates [54] (Figure S3). Over time, both NPM1_41–65_ and NPM1_107–120_ exhibited increasing profiles of ThT fluorescence, even if with great differences in the kinetic, as evaluable from the comparison of t_1/2_ values that represent the time values at which fluorescence intensity reaches its maximum value/2. Indeed, while NPM1_41–65_ appeared to aggregate quickly (Figure 2A) with a t_1/2_ = 5 min and a subsequent net decrease in signal due to fibrillization in 3 h, NPM1_107–120_ presented longer times of aggregation (Figure 4A) with a t_1/2_ = 20 min and the total abolishment of the fluorescence signal, in ~20 h. Both sequences present a negative net charge at neutral pH—fragment 41–65 with a double charge with respect to 107–120 (Table 1). Consistently, the presence of two positive residues only in NPM1_41–65_ (Arg^45^ and Lys^54^) could explain the higher speed of aggregation.

The conformational preferences of these peptides were analyzed over time through CD spectroscopy, and deconvolution data are reported in Table 2. As expected, all three peptides at t = 0 exhibited a mixture of conformation, with a prevalence of the β-structure, but the evolution over time was peculiar for each sequence. Indeed, while NPM1_41–65_ showed a stable profile over time (Figure 2B), NPM1_107–120_ presented a slight increase in the β-structure at the expense of helical content and a decrease in the Cotton effect, starting from 2 h (Figure 4B). More markedly, NPM1_69–83_ exhibited a transition toward the β-sheet in 2 h (up to ~50% of beta) (Figure 3B, Table 2), allowing the formation of a well-defined secondary structure despite the presence of Pro^71^, which is often reported to interrupt secondary structures [60].

A few peptides were also analyzed by 1D [^1^H] and 2D [^1^H, ^1^H] NMR spectroscopy at different times. For the 69–83 fragment, the comparison of 1D spectra of freshly prepared sample (t = 0) and after 4 h and 2 days (Figure S4A) did not show a chemical shift and/or intensity changes, as also evident from the overlay of 2D TOCSY spectra acquired at t = 0 and 4 days (Figure S4B). The 2D NOESY 300 spectrum (Figure S4C) contained almost solely diagonal peaks and pointed out extended/random conformations typical of a low molecular weight species.

The SEM analyses of all peptides were carried out at two different times of aggregation, 0 and 24 h. The SEM images of NPM1_41–65_ at both times (Figure S5A–C and Figure 2C–E) show the presence of amorphous aggregates, while for NPM1_107–120_ (Figure S6A–C and Figure 4C–E), partially mature fibers are visible only after 24 h of aggregation. For both sequences, in the analyzed interval time, the population of compact structures is very low or negligible.

Conversely, NPM1_69–83_ displayed amyloid fibers “in formation” at t 0 (Figure S5D–F) and mature at 24 h (Figure 3C–E), with an average length of (4.6 ± 0.5) × 10 µm and a diameter of (4.10 ± 0.16) µm (Figure 3D).

- Random region: ThT assay, conformational and SEM analyses of NPM1_84–93._

**Figure 5:**
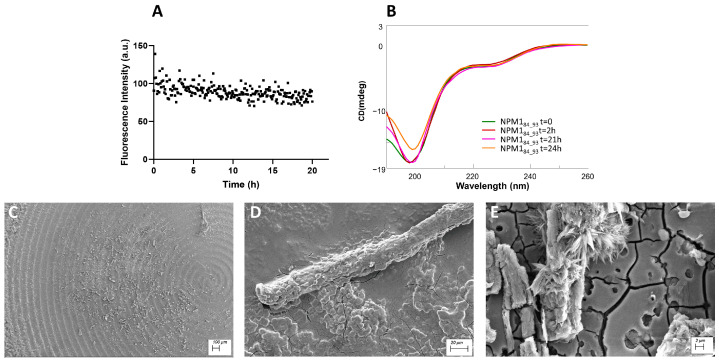
NPM1_84–93_ characterization: (**A**) Time course of ThT fluorescence emission intensity; (**B**) CD spectra over time; (**C**–**E**) SEM micrographs registered at 24 h at 100, 20 and 2 µm.

With similar assays and times of acquisition, the random fragment 84–93 was investigated. This sequence appeared completely unable to bind ThT (Figure 5A), since no signal variation was detected over 20 h (Figure S3) and in a CD analysis (Figure 5B), confirming a prevalent random content mixing with α-helix, which did not change over time (Table 2). In agreement with CD data, the comparison of 1D spectra indicated no conformational changes between t = 0 and t = 3 d (Figure S7A), and in the 2D NOESY 300 spectrum (Figure S7B), the lack of contacts outside the diagonal peaks confirmed the absence of peptide structuration.

Coherently, a SEM analysis of NPM1 84–93 did not evidence the amyloid features of a few unripe fibers (Figure S6D–F), which appeared mainly still in formation, even at 24 h (Figure 5C–E).

- Helical regions: ThT assay, conformational and SEM analyses of NPM1_127–139_ and NPM1_242–259._

**Figure 6:**
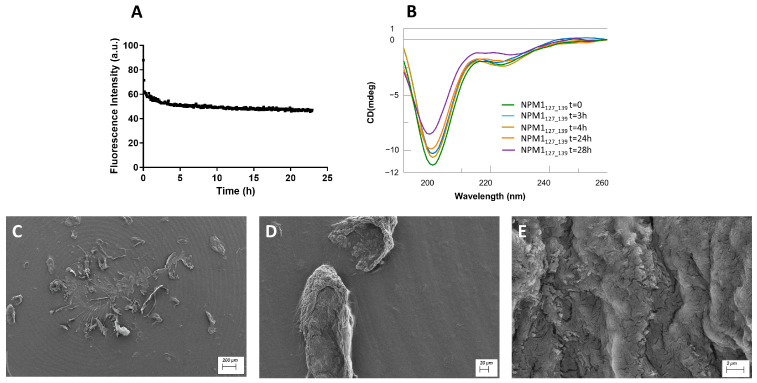
NPM1_127–139_ characterization: (**A**) Time course of ThT fluorescence emission intensity; (**B**) CD spectra over time; (**C**–**E**) SEM micrographs registered at 24 h at 100, 20 and 2 µm.

**Figure 7:**
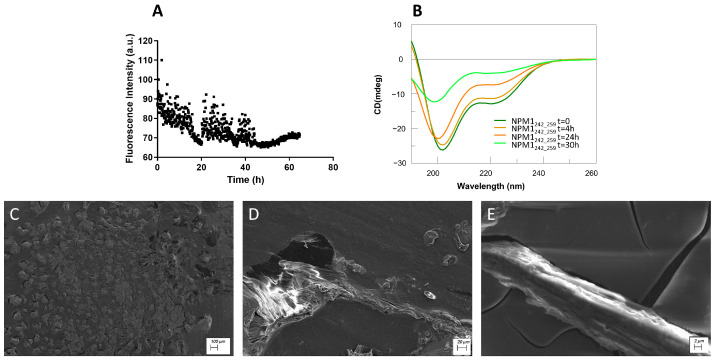
NPM1_242–259_ characterization: (**A**) Time course of ThT fluorescence emission intensity; (**B**) CD spectra over time; (**C**–**E**) SEM micrographs registered at 24 h at 100, 20 and 2 µm.

The 127–139 and 242–259 fragments presented a similar behavior in the ThT fluorescence assay. Both revealed an inability to bind the amyloid dye, even at long times of stirring (Figure 6A, Figure 7A and Figure S3). As expected, the 242–259 fragment presented a good helical content, especially at t = 0 of aggregation (Figure 7B, Table 2), which, after 30 h, was partially lost. Conversely, NPM1_127–139_, differently from the prediction, exhibited a prevalent random state that persisted for 28 h of analysis (Figure 6B, Table 2).

In the SEM analysis, NPM1_127–139_ showed the formation of a dense network of fibers [61] already at t = 0. Using higher magnification, it was possible to observe how the fibers tend to associate themselves in the form of wide ribbons or bundles (Figure S8A–C). In detail, these bundles appeared thin (2.3 ± 1.0 µm) in diameter and in length (9.0 ± 4 × 10 µm) (Figure S8B), but after 24 h, they disassembled to form insoluble aggregates (Figure 6C–E). On the other hand, poor aggregation propensity was found for the peptide NPM1_242–259_ (Figure S8D–F and Figure 7C–E), whose aggregates were unable to evolve toward amyloid fibers.

- Cellular effects of NPM1 fragments

**Figure 8:**
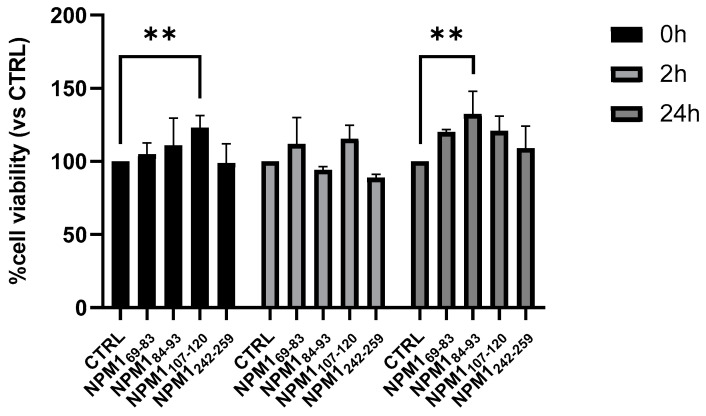
Cell viability effects of NPM1 fragment. The histogram reports the percent of cell viability (100% viable cells represent the control, CTRL) treated with peptides pre-incubated at three different times: 0, 2 and 24 h. The histogram is representative of a single experiment performed in triplicate. Results are expressed as mean ± SD. The statistical analyses were performed with the GraphPad Prism 9 software using two-way ANOVA corrected for multiple comparison by the Dunnet test (** *p* < 0.005).

With the aim to evaluate the potential toxic effects of NPM1 fragments [62], several designed peptides were analyzed in a cell viability assay employing SHSY5 cells at different times of aggregation. From the MTT assay reported in Figure 8, none of the NPM1 fragments turned cytotoxic, while a slight increase in cell viability was observed at t = 0 h only for NPM1_107–120_, whereas no statistically significant effect was observed for NPM1_69–83_.

## 3. Materials and Methods

### 3.1. Peptide Synthesis

The reagents for solid-phase peptide synthesis (SSPS) were purchased from Iris Biotech (Marktredwitz, Germany) and the solvents for HPLC analyses from Romil (Dublin, Ireland). All peptides were chemically synthesized following Fmoc solid-phase peptide synthesis protocols, purified by RP-HPLC and identified through LC-MS. The peptides were pre-treated overnight with hexafluoro-2-propanol (HFIP), lyophilized and stored at −20 °C until use.

### 3.2. Far-UV CD Spectroscopy

The samples were prepared by dilution of freshly prepared stock solutions (1 mM peptide, on average). CD spectra were recorded on a Jasco J-815 spectropolarimeter (JASCO, Tokyo, Japan) at 25 °C in the far-UV region from 190 to 260 nm in a 0.1 cm quartz cuvette. The other experimental settings were: 20 nm/min scan speed, 2.0 nm band width, 0.2 nm resolution, 50 mdeg sensitivity and 4 s response. Each spectrum was obtained averaging three scans, subtracting contributions from the corresponding scans. The peptide concentrations were 100 µM for all fragments and 200 µM for NPM1_69–83_ in 10 mM phosphate buffer, pH 7.4. Deconvolutions of CD spectra were obtained by BESTSEL software (http://bestsel.elte.hu/) [63].

### 3.3. ThT Assay

ThT assays were performed in 50 mM phosphate buffer, at 25 °C, using a ThT concentration of 50 µM. The peptide concentrations were based on a compromise between solubility and fluorescence signal: 470 µM for NPM1_41–65_, 200 µM for NPM1_107–120,_ 400 µM for NPM1_127–139_, 800 µM for NPM1_69–83,_ 800 µM for NPM1_84–93_ and 800 µM for NPM1_242–259_. ThT fluorescence was measured using a Jasco (Japan, Tokyo) FP 8300 spectrofluorometer with a 10 mm path-length quartz cuvette, under magnetic stirring. Spectra were collected every 5–15 min, using excitation at 440 and emission at 483 nm. Fluorescence intensities were subtracted from ThT alone.

### 3.4. SEM Analysis

NPM1 peptides were analyzed by SEM microscopy, as already reported [59]. All peptides, except NPM1_107–120_ (200 μM), were dissolved at 800 μM in 50 mM phosphate buffer at pH 7.4 and analyzed at t 0 and under stirring after 24 h. In detail, samples were dropped on a typical SEM stub and gold-sputtered at 20 nm thickness with the HR208 Cressington sputter coater and analyzed at 5–10 kV with an SE2 detector by Ultra Plus FESEM scanning electron microscope (Zeiss, Oberkochen, Germany).

### 3.5. NMR Experiments

NMR spectra were registered at a temperature of 25 °C on a Varian Unity Inova 600 MHz NMR spectrometer provided with a cold probe. For NMR sample preparation, the peptides were dissolved in a total volume of 540 μL, including 500 μL of 10 mM NaP buffer and 40 μL of D_2_O (Deuterium Oxide, 98% D, Sigma-Aldrich, Milan, Italy). Both NPM1_69–83_ and NPM1_84–93_ were analyzed at 300 μM concentration and pH values equal to 7.4 and 7.2, respectively. Briefly, the following NMR spectra were acquired: 1D [^1^H] (with 128 scans), 2D [^1^H, ^1^H] TOCSY [64] (70 ms mixing time, 16 scans, 256 FIDs, 1024 data points in t2) and 2D [^1^H, ^1^H] NOESY spectra [65] (300 ms mixing time, 128 scans, 512 FIDs, 2048 data points). For the NPM1_69-83_ peptide, the 1D [^1^H] spectra were recorded for the freshly prepared sample (t = 0), and after 4 h (t = 4 h) and 2 days (t = 2 d) after sample preparation, whereas 2D [^1^H, ^1^H] TOCSY spectra were recorded at t = 0 and after 4 days (t = 4 d). For the NPM1_84–93_ peptide, 1D [^1^H] spectra were recorded at t = 0, and after 4 h (t = 4 h) and 3 days (t = 3 d). The 2D [^1^H, ^1^H] NOESY spectra were also acquired for both peptides 4 days after sample preparation. The NMR samples were stored at 4 °C in between the experiments registered at different times. Water suppression was obtained by *Excitation Sculpting.* The software VNMRJ 1.1D (Varian, Italy) was used for spectra processing; NEASY [66], included in CARA (http://cara.nmr.ch/doku.php), and UCSF sparky [67] were employed for spectra analyses. The water signal was implemented for chemical shifts referencing (4.75 ppm).

### 3.6. Cells

SH-SY5Y cells were grown at 37 °C in a humidified atmosphere of 5% CO_2_ in DMEM (Dulbecco’s Modified Eagle Medium GIBCO, Paisley, UK), supplemented with 10% heat inactivated fetal bovine serum (FBS) (GIBCO), 2 mM L-glutamine, 50 ng/mL streptomycin and 50 units/mL penicillin.

### 3.7. Cell Viability

NPM1_69–83_, NPM1_84–93_, NPM1_107–120_ and NPM1_242–259_ peptides were tested using a 1.6 mM stock solution and were pre-incubated to allow aggregation at different time points: 0, 2 and 24 h. The peptides were assayed at the final concentration of 400 μM.

Cells were seeded in triplicate in 96-well plates at a density of 7500 cells/well. Cells were incubated with the peptides at the time points mentioned above for 24 h at 37 °C in a humidified atmosphere of 5% CO_2_. In the last 4 h of incubation, 3-(4,5-dimethylthiazol-2-yl)-2,5-diphenyltetrazolium bromide (MTT) was added to the cells. DMSO was then added to allow the reduction of MTT into formazan crystals by living cells, as previously reported [68,69]. The absorbance was measured at 560 nm by Glomax^®^ Discover Microplate Reader (Promegam Madison, WI, USA).

## 4. Conclusions

NPM1 is at the center of a wide and crucial interactome, which becomes dysregulated in AML cells [70]. Hence, a deep analysis of the structural factors involved in leukemogenesis is of utmost importance. Our recent investigations correlate, directly and undoubtedly, the aggregation to AML mutations occurring in the third helix of the CTD. Starting from the importance of the mutual influence among domains in NPM1 [45], herein, we analyzed the presence of APRs within the entire protein through the combination of theoretical and experimental procedures. On the basis of the prediction of aggregation propensity, we designed several peptides covering different protein regions located almost completely outside the CTD. By analyzing the fragments located in the β-structure regions, we observed the most evident conformational plasticity eventually prone to aggregation. Indeed, the 41–65, 69–83 and 107–120 fragments demonstrated the ability to bind ThT even with different kinetics of aggregation that depend on their aminoacidic composition and that can, in turn, explain the SEM results. In detail, sequence 69–83 demonstrated greater conformational transitions in the CD analysis and appeared bound to ThT at t = 0 of the analysis. It provided fibers with a defined amyloid character even at t = 0. Conversely, the fast binding to ThT exhibited by the 41–65 fragment only led to amorphous aggregation, likely due to the short times of organization of the peptide chains. On the other hand, the slow aggregation exhibited by 107–120 caused the formation of unripe fibers even after 24 h of aggregation. Noticeably, all the other investigated NPM1 protein regions did not exhibit ThT binding and presented poor conformational variations over time. In detail, the random 84–93 fragment provided a typical CD profile, constant over time, and immature fibers at SEM analysis; the 242–259 fragment was also confirmed as a stable helix with no amyloid evolution. The overall data unveil the presence of hot spots of aggregation, mostly in the NTD, but none of the identified APRs demonstrated a well-defined amyloid character causing cytotoxicity in SHSY5, as instead demonstrated by other NPM1 stretches [46]. In conclusion, even if further experiments on the potential synergy of aggregation of the entire AML-mutated protein are required, the presented data allow adding new gussets in the puzzled way of molecular determinants of cytoplasmatic accumulation of NPMc+ and could introduce innovative therapeutic strategies targeting the NPM1-AML subtype.

## Figures and Tables

**Table 1:** NPM1 fragments analyzed in this study: peptide sequences, pIs, conformation in NPM1 structure.

Fragments	Sequences	pI	Net Charge at Neutral pH	Conformation
41–65	Ac-Q^41^LSLRTVSLGAGAKDELHIVEAEAM^65^-NH_2_	4.52	−1.9	β-sheet
69–83	Ac-G^69^SPIKVTLATLKMSV^83^-NH_2_	14	2	β-sheet
84–93	Ac-^84^QPTVSLGGFE^93^-NH_2_	0	−1	random
107–120	Ac-G^107^PVHISGQHLVAVE^120^-NH_2_	6.05	−0.8	β-sheet
127–139	Ac-D^127^EEEEDVKLLSIS^139^-NH_2_	3.27	−5	α-helix
242–259	Ac-S^242^SVEDIKAKMQASIEKGG^259^-NH_2_	7.36	0	α-helix

**Table 2:** Deconvolution of CD spectra of all peptides at indicated times.

	Time (h)	Helix	Beta	Turn	Others
NPM1_41–65_	0	0.0	30.8	25.5	43.7
	2	0.1	27.9	21.7	50.3
	21	0.6	28.2	21.0	50.3
NPM1_69–83_	0	9.1	35.3	13.6	42.0
	2	3.6	49.7	9.9	36.8
	4	0.0	34.9	18.5	46.7
	30	0.0	35.4	17.9	46.8
NPM1_84–93_	0	7.0	27.3	22.9	42.7
	2	5.7	25.6	20.4	48.2
	21	7.2	27.3	21.8	43.7
	24	7.0	24.3	19.9	48.8
NPM1_107–120_	0	5.8	25.4	19.7	49.1
	2	3.9	27.9	19.1	49.1
	4	2.9	31.2	18.2	47.7
	28	2.5	34.3	17.2	45.9
	30	0.2	36.4	16.0	47.4
NPM1_127–139_	0	0.5	32.3	19.5	47.6
	3	0.0	32.7	19.4	47.9
	4	0.0	33.2	19.4	47.4
	24	0.0	34.2	19.1	46.7
	28	0.0	34.6	18.8	46.6
NPM1_242–259_	0	11.5	24.1	16.8	47.6
	4	9.0	25.5	17.3	48.2
	24	3.5	29.6	18.0	48.9
	30	0.1	35.3	16.9	47.7

## Supplementary Materials

The following supporting information can be downloaded at: https://www.mdpi.com/article/10.3390/ijms232314704/s1.
