# Supplementary material for: Insights into Network of Hot Spots of Aggregation in Nucleophosmin 1
Source: Int J Mol Sci. 2022 Nov 25;23(23):14704. doi: 10.3390/ijms232314704 (PMC9736328; doi:10.3390/ijms232314704)
Supplement: Supplementary file 1 [file ijms-23-14704-s001.zip › ijms-2035977-supplementary.pdf]

Supporting information:

ARTICLE

Insights into network of hot-spots of aggregation in Nucleophosmin 1

Daniele Florio[1#], Sara La Manna[1#], Concetta Di Natale[2], Marilisa Leone[3], Flavia Anna Mercurio[3], Fabiana Napolitano[4], Anna Maria Malfitano[4] and Daniela Marasco[1,3*]

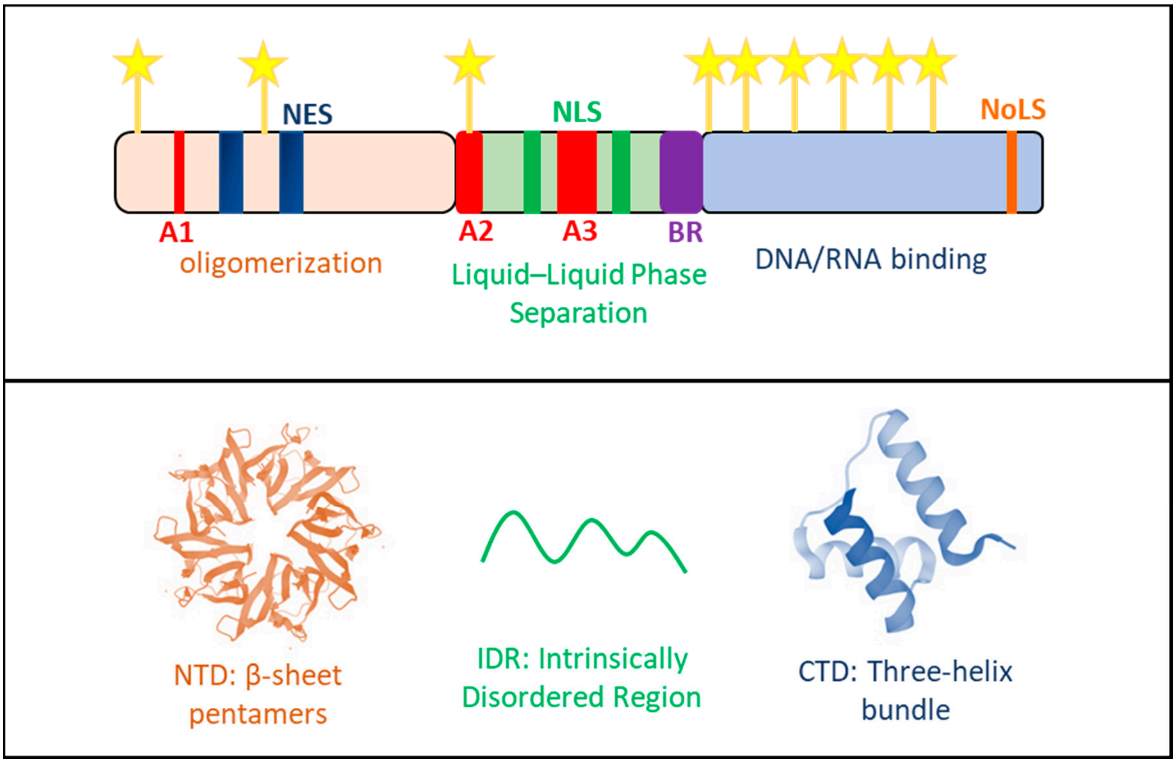

Figure S1. Structure of NPM1 and functional domains. Upper panel: Common modular partition of NPM1 (A1-A3 Acidic, BR Basic Regions, NoLS nucleolar localization sequence, NLS Nuclear Localization, NES Nuclear Export Signal) and post-translational modifications (indicated with stars). Lower panel: tertiary structures of corresponding domains: X-ray structure of NTD (residues 1-117) [1] (left), three-helix bundle of the CTDwt (residues 243-294) studied by NMR spectroscopy[2] (right). The central IDR (residues 118-242) portion is predicted mainly as a disordered region (middle).

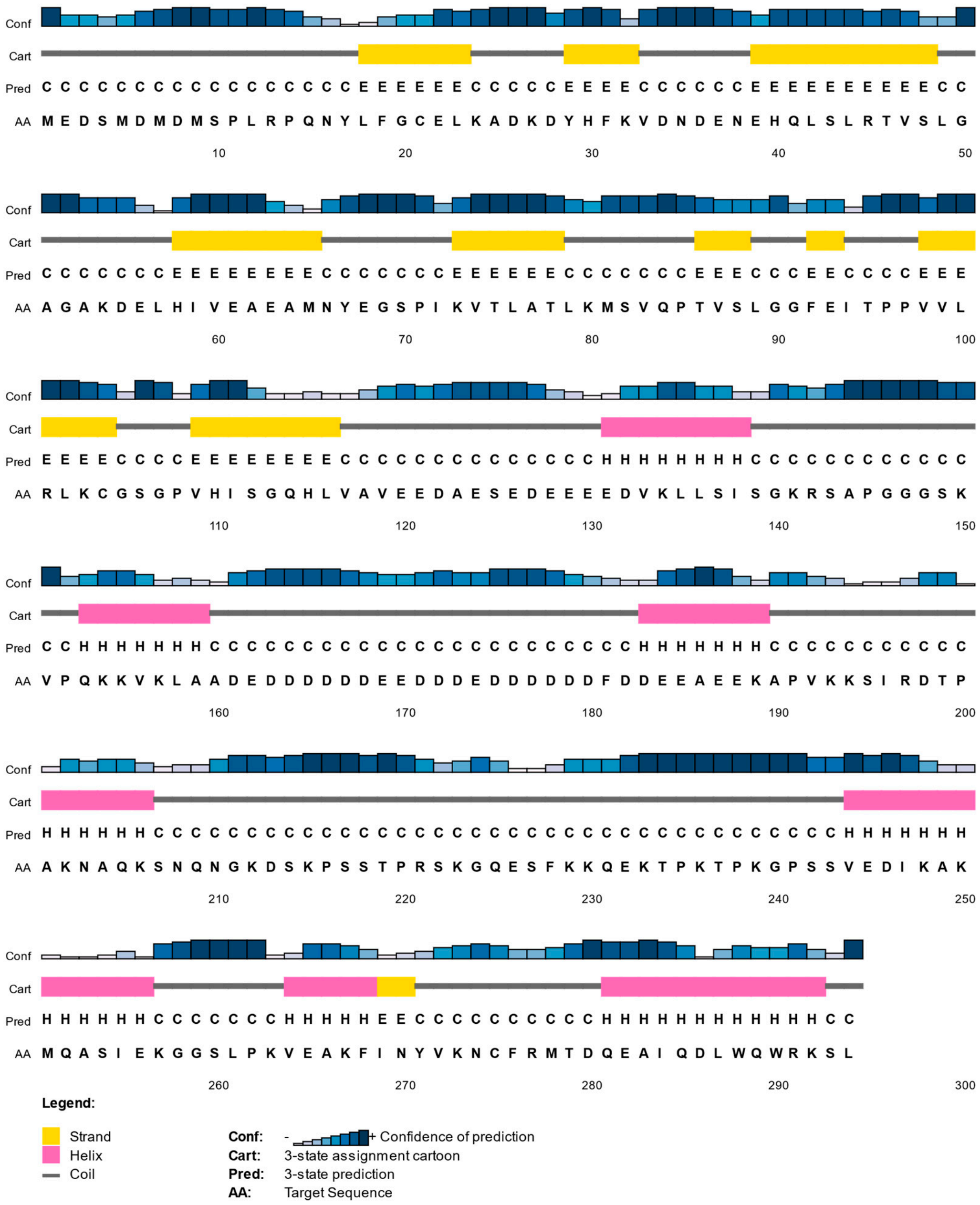

Figure S2. Prediction of NPM1 secondary structure elements obtained by using the PSIPRED server.

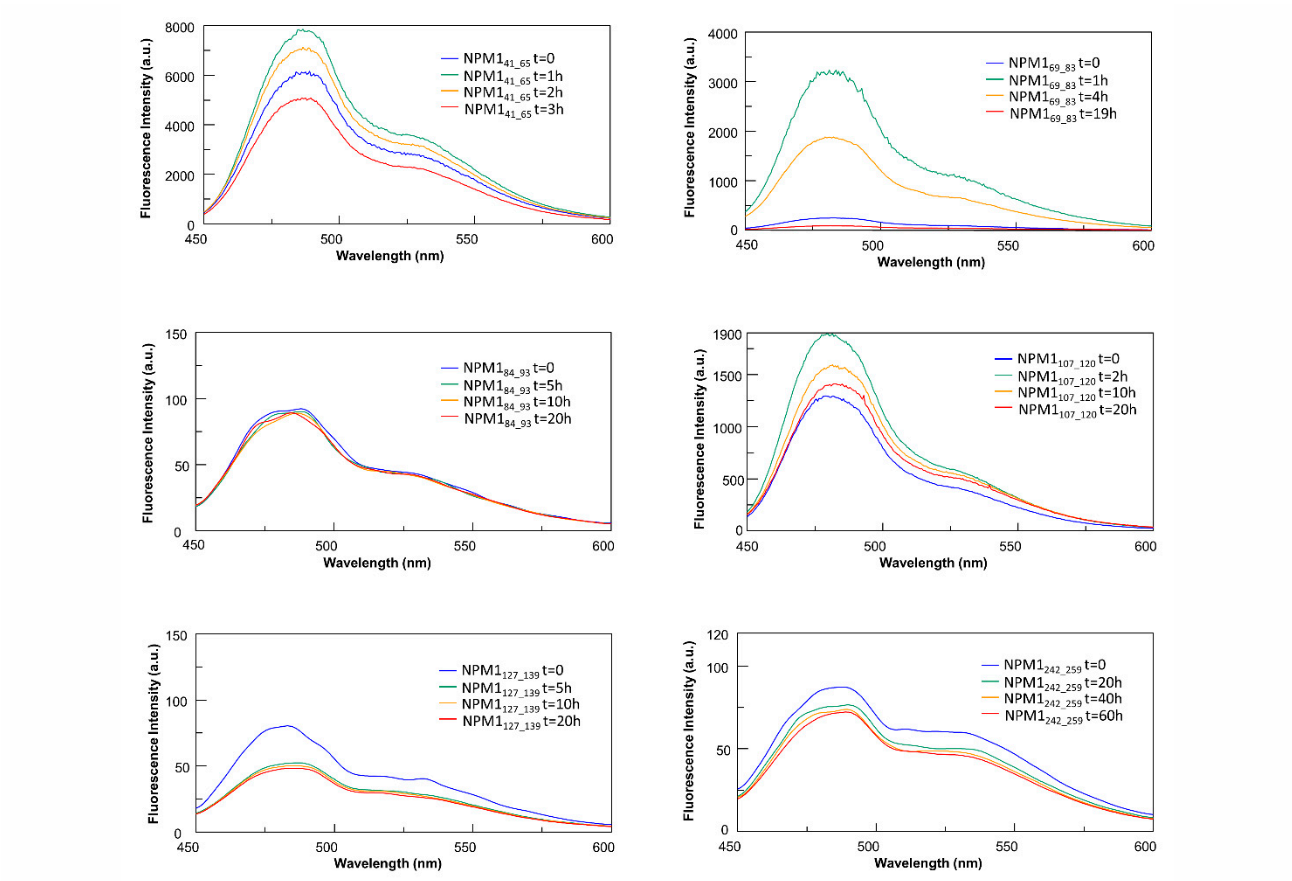

Figure S3. ThT fluorescence spectra of all peptides at indicated times.

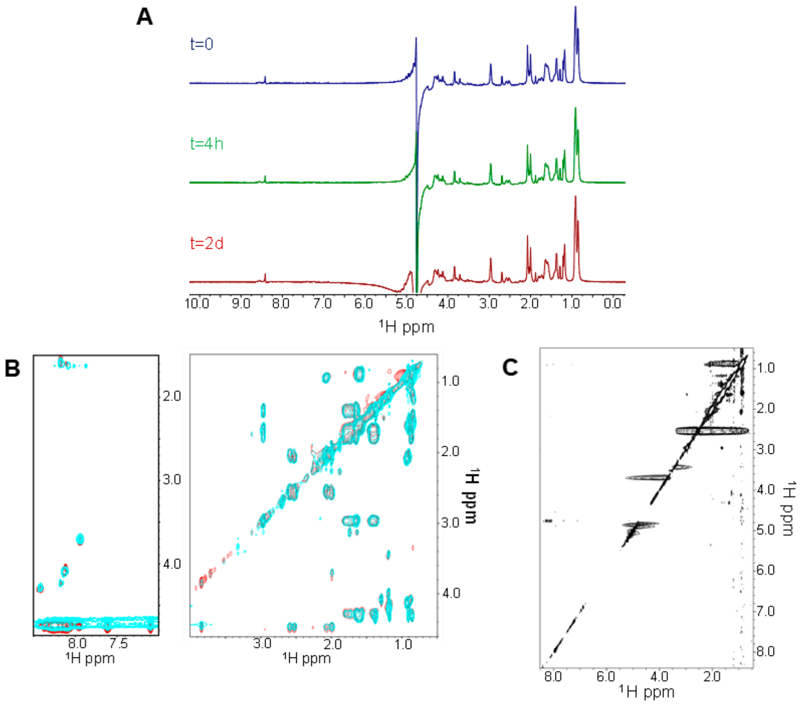

Figure S4. NMR investigations of NPM1$_{69-83}$: (A) the comparison of 1D spectra registered with freshly prepared sample (t=0), after 4 hours (t=4h) and 2 days (t=2d). (B) 2D TOCSY spectra acquired at t=0 (red) and t=4d (cyan). (C) 2D NOESY 300 spectrum.

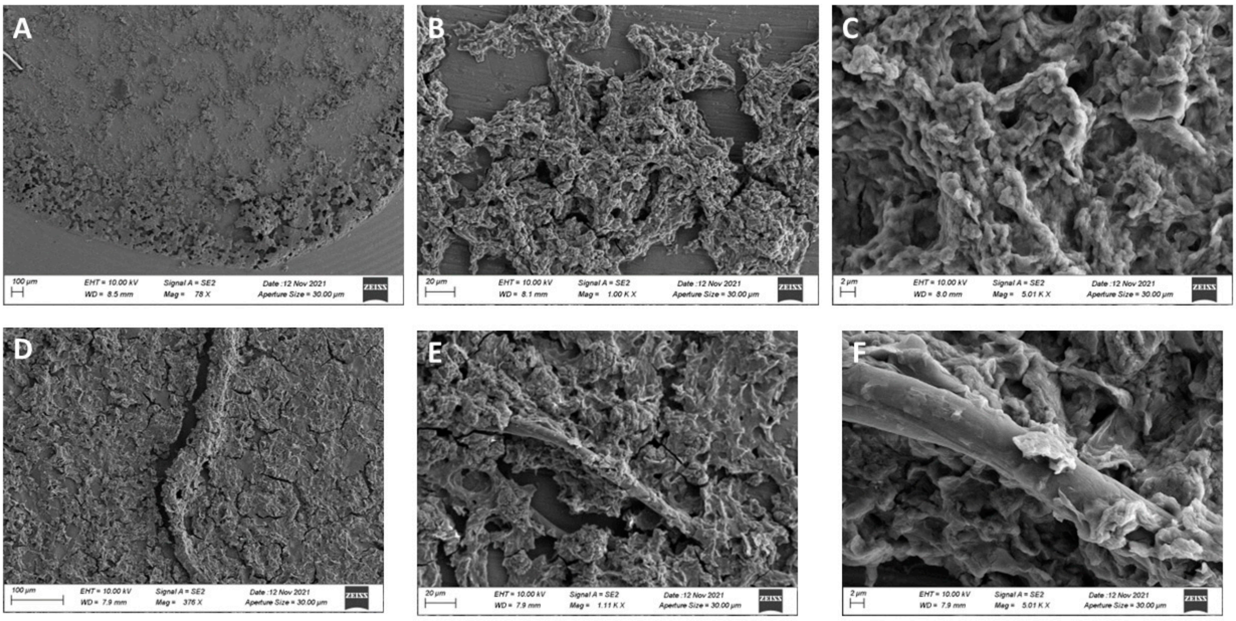

Figure S5. SEM micrographs of NPM1$_{41-65}$ (upper panel) NPM1$_{69-83}$ (lower panel) at t=0h at 100 (A,D), 20 (B,E) and 2 (C,F) μm.

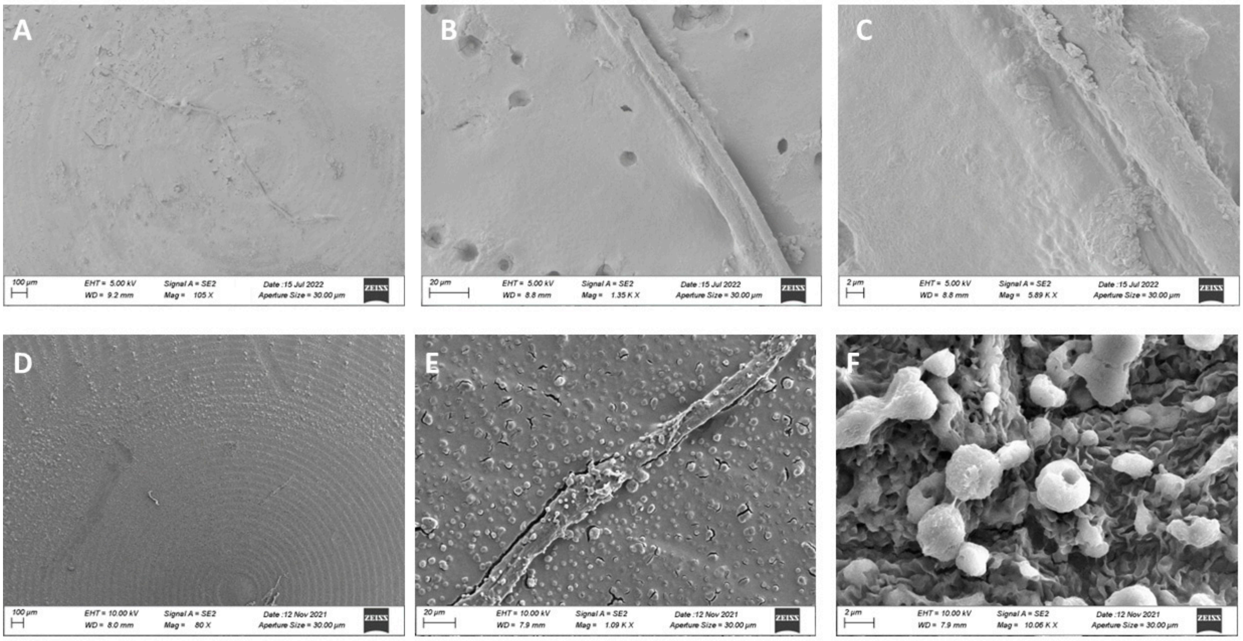

Figure S6. SEM micrographs of NPM1$_{107-120}$ (upper panel) NPM1$_{84-93}$ (lower panel) registered at 0h at 100 (A,D), 20 (B,E) and 2 (C,F) μm.

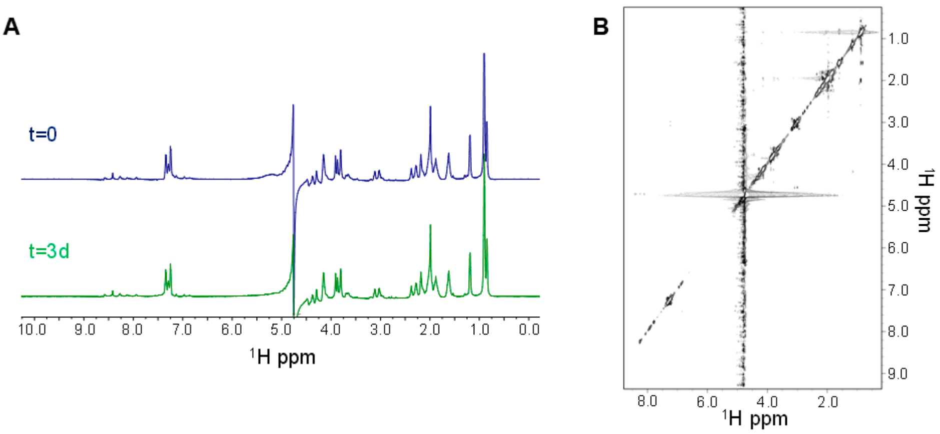

Figure S7. NMR investigations of NPM1$_{84-93}$: (A) the comparison of 1D spectra registered with freshly prepared sample (t=0) and after 3 days (t=3d). (B) 2D NOESY 300 spectrum.

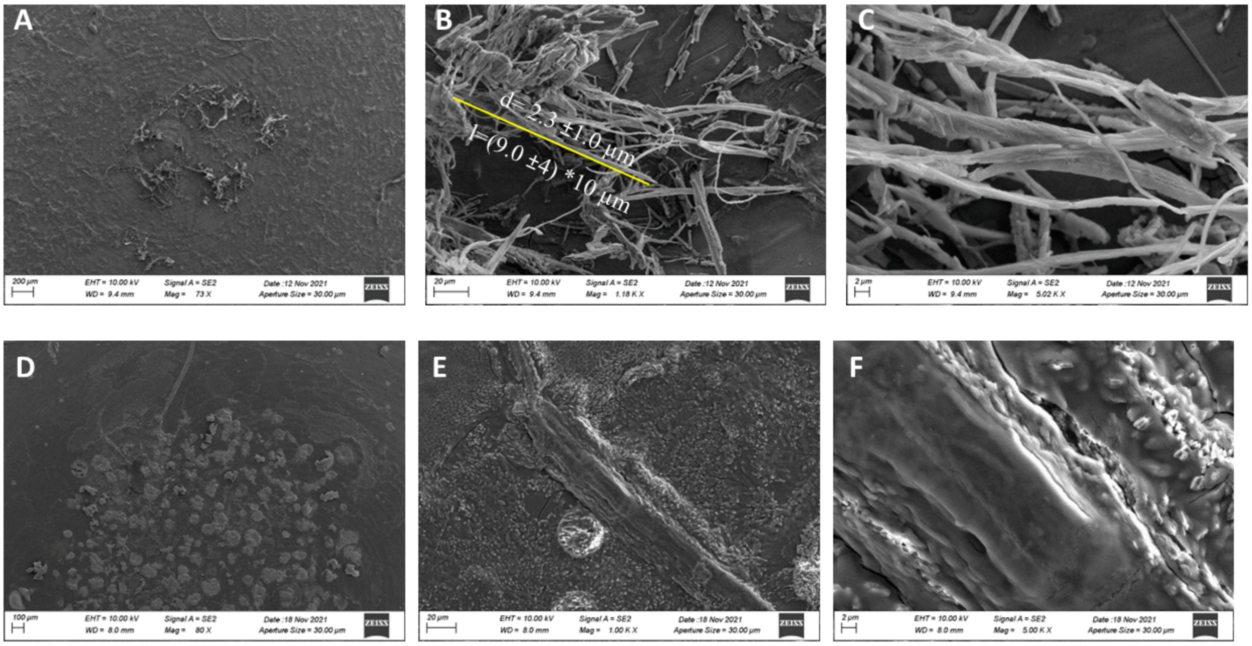

Figure S8. SEM micrographs of NPM1$_{127-139}$ (upper panel) NPM1$_{242-259}$ (lower panel) at t=0h at 100 (A,D), 20 (B,D) and 2 (C,E) µm.
